# Supplementary material for: Cathelicidin-3 Associated With Serum Extracellular Vesicles Enables Early Diagnosis of a Transmissible Cancer
Source: Front Immunol. 2022 Mar 29;13:858423. doi: 10.3389/fimmu.2022.858423 (PMC9004462; doi:10.3389/fimmu.2022.858423)
Supplement: Supplementary file 1 [file Presentation_1.pdf]

Supplementary Material

Supplementary Tables for *Cathelicidin-3 associated with serum extracellular vesicles enables early diagnosis of a transmissible cancer*.

This file contains Supplementary tables 2–4

Supplementary table 2. EV proteins accuracy for the diagnosis of DFT1 in the discovery cohort with an area under the curve (AUC) ≥ 0.9. All numbers for AUC, sensitivity, specificity, and accuracy are in fractions. 95% confidence intervals.

Protein name	AUC	Specificity	Sensitivity	Accuracy	UniProt
**CATH3**	1.000 (1.000, 1.000)	1.000 (1.000, 1.000)	1.000 (1.000, 1.000)	1.000 (1.000, 1.000)	G3W0S2
**CTGF**	1.000 (1.000, 1.000)	1.000 (1.000, 1.000)	1.000 (1.000, 1.000)	1.000 (1.000, 1.000)	G3WBU2
**C5**	1.000 (1.000, 1.000)	1.000 (1.000, 1.000)	1.000 (1.000, 1.000)	1.000 (1.000, 1.000)	G3WZ02
**C8G**	0.992 (0.950, 1.000)	1.000 (0.900, 1.000)	0.917 (0.833, 1.000)	0.955 (0.900, 1.000)	G3W1Q6
**SPP2**	0.983 (0.925, 1.000)	1.000 (0.900, 1.000)	0.917 (0.833, 1.000)	0.955 (0.900, 1.000)	G3VQP6
**C6**	0.983 (0.925, 1.000)	1.000 (0.800, 1.000)	0.917 (0.833, 1.000)	0.955 (0.909, 1.000)	G3WFL6
**C7**	0.983 (0.925, 1.000)	1.000 (0.800, 1.000)	0.917 (0.833, 1.000)	0.955 (0.909, 1.000)	G3WK50; G3WK51
**F10**	0.983 (0.925, 1.000)	1.000 (0.800, 1.000)	0.917 (0.833, 1.000)	0.955 (0.908, 1.000)	G3WXN7
**C8A**	0.975 (0.900, 1.000)	1.000 (0.900, 1.000)	0.917 (0.750, 1.000)	0.955 (0.864, 1.000)	G3VVQ8; G3VVQ9
**ALDOA**	0.975 (0.900, 1.000)	1.00 (0.800, 1.000)	0.833 (0.750, 1.000)	0.900 (0.864, 1.000)	G3WAK9
**C9**	0.967 (0.900, 1.000)	1.000 (0.700, 1.000)	0.833 (0.750, 1.000)	0.909 (0.864, 1.000)	G3W3E7
**ACTN1**	0.958 (0.867, 1.000)	1.000 (0.800, 1.000)	0.833 (0.750, 1.000)	0.909 (0.810, 1.000)	G3WFU1; G3WFU2
**CPNE1**	0.958 (0.867, 1.000)	0.800 (0.700, 1.000)	1.000 (0.750, 1.000)	0.909 (0.818, 1.000)	G3X2P2
**PLEK**	0.950 (0.825, 1.000)	1.000 (1.000, 1.000)	0.917 (0.750, 1.000)	0.955 (0.864, 1.000)	G3VFV4; G3VFV5
**VCP**	0.942 (0.817, 1.000)	1.000 (0.900, 1.000)	0.833 (0.667, 1.000)	0.909 (0.818, 1.000)	G3WW22
**IGA***	0.933 (0.808, 1.000)	0.800 (0.600, 1.00)	0.917 (0.667, 1.000)	0.864 (0.773, 1.000)	G3VS26
**BIN2**	0.925 (0.783, 1.000)	0.900 (0.800, 1.000)	0.917 (0.667, 1.000)	0.909 (0773, 1.000)	G3VUC7
**LOC100931899**	0.925 (0.792, 1.000)	0.900 (0.700, 1.000)	0.833 (0.583, 1.000)	0.864 (0.773, 1.000)	G3VBX5
**PFN1**	0.925 (0.792, 1.000)	0.900 (0.700, 1.000)	0.917 (0.667, 1.000)	0.909 (0.773, 1.000)	G3VU42
**YWHAZ**	0.925 (0.766, 1.000)	0.900 (0.700, 1.000)	1.000 (1.000, 1.000)	0.955 (0.864, 1.000)	G3WHE0
**TGFBI**	0.917 (0.767, 1.000)	1.000 (0.700, 1.000)	0.750 (0.583, 1.000)	0.864 (0.773, 1.000)	G3VMG6
**IGH***	0.917 (0.775, 1.000)	0.800 (0.600, 1.000)	0.917 (0.583, 1.000)	0.864 (0.773, 1.000)	G3VVK2
**PTN**	0.908 (0.758, 1.000)	1.000 (0.600, 1.000)	0.750 (0.583, 1.000)	0.864 (0.773, 1.000)	G3VRT4; G3VRT5

**ANXA11**	0.908 (0.767, 1.000)	0.800 (0.600, 1.000)	0.917 (0.583, 1.000)	0.864 (0.773, 1.000)	G3VJ37; G3VJ38
**CAPZB**	0.908 (0.750, 1.000)	0.900 (0.700, 1.000)	0.917 (0.667, 1.000)	0.909 (0.773, 1.000)	G3VTU6
**LOC100924827**	0.908 (0.767, 1.000)	0.900 (0.600, 1.000)	0.750 (0.583, 1.000)	0.818 (0.773, 1.000)	G3VT38
**TLN1**	0.908 (0.758, 1.000)	0.900 (0.700, 1.000)	0.833 (0.583, 1.000)	0.864 (0.773, 1.000)	G3WN56
**CAPZA2**	0.900 (0.750, 1.000)	0.900 (0.600, 1.000)	0.833 (0.583, 1.000)	0.864 (0.773, 1.000)	G3WVH2; G3WVH3
**MPO**	0.900 (0.750, 1.000)	1.000 (0.600, 1.000)	0.750 (0.583, 1.000)	0.864 (0.773, 1.000)	G3WE24
**ANXA4**	0.908 (0.758, 1.000)	1.000 (0.800, 1.000)	0.750 (0.583, 1.000)	0.864 (0.773, 1.000)	G3WNV1; G3WNV2
**ACTR3**	0.900 (0.750, 1.000)	0.700 (0.600, 1.000)	1.000 (0.583, 1.000)	0.864 (0.773, 1.000)	G3WIY5

*Proteins blasted against the Tasmanian devil reference genome (GCA_902635505.1 mSarHar1.11) using the online NCBI protein Basic Local Alignment Search Tool (BLAST).

Supplementary table 3. EV proteins accuracy for the diagnosis of DFTD in the validation cohort with an area under the curve (AUC) ≥ 0.9. All numbers for AUC, sensitivity, specificity, and accuracy are in fractions. 95% confidence intervals.

Protein name	AUC	Specificity	Sensitivity	Accuracy	UniProt number
**CATH3**	0.925 (0.840, 0.989)	0.941 (0.882, 1.000)	0.879 (0.727, 0.97)	0.900 (0.820, 0.980)	G3W0S2
**PFN1**	0.918 (0.804, 0.995)	0.882 (0.765, 1.000)	0.909 (0.788, 1.000)	0.900 (0.820, 0.98)	G3VU42
**ILK**	0.914 (0.822, 0.980)	0.824 (0.647, 1.000)	0.848 (0.697, 1.000)	0.840 (0.760, 0.960)	G3W679
**LIMS1**	0.909 (0.818, 0.973)	0.765 (0.646, 1.000)	0.909 (0.636, 1.000)	0.860 (0.740, 0.960)	G3VJH5

Supplementary table 4. Kendall correlation of proteins with tumour volumes. DFTD infected animals from the discovery and validation dataset (n=45). P values were adjusted by the Benjamini-Hochberg method.

Protein name	Kendall's tau	Corrected P value	Uniprot number
MYH10	0.42	0.001	G3W344
TGFBI	0.41	0.001	G3VMG6
CTGF	0.41	0.001	G3WBU2
GADPH	0.36	0.004	G3WQA5
HSPA5	0.36	0.004	G3W4H8
ANXA4	0.36	0.004	G3WNV1;G3WNV2
MYL6	0.35	0.006	G3VPK8
LOC100924827	0.34	0.006	G3VT38
MYH9	0.34	0.006	G3W8S4
ITGA2B	0.34	0.008	G3WU27
RAP1A	0.33	0.008	G3WME1
TNN	0.33	0.008	G3WK59
FGL1	0.33	0.008	G3W2N2
RHOA	0.33	0.008	G3WUV1
PPIA	0.33	0.008	G3WXF8
LOC105750812	0.32	0.010	G3VPL9
LOC100931899	0.31	0.012	G3VBX5
PTN	0.31	0.012	G3VRT4; G3VRT5
IGA*	0.30	0.017	G3VS26
ACTB	0.29	0.018	G3WHH2; G3WP12
TUBB2A	0.29	0.019	G3WMM3
YWHAQ	0.29	0.020	G3VS43
ANXA1	0.28	0.022	G3W8D2
C5	0.28	0.022	G3WZ02
LOC100932693	-0.28	0.022	G3WEF1
DCN	0.27	0.029	G3VVA7

CCDC3	0.27	0.030	G3WLM3
DSTN	0.27	0.032	G3W5M4
LIMS1	0.27	0.032	G3VJH5; G3VJH6
SELP	0.27	0.032	G3W7A0
CFL1	0.26	0.034	G3VHS2
CAPZA2	0.26	0.035	G3WVH2;
CAPZB	0.26	0.037	G3VTU6
YWHAZ	0.26	0.037	G3WHE0
ARHGDIB	0.26	0.039	G3WV18

*Proteins blasted against the Tasmanian devil reference genome (GCA_902635505.1 mSarHar1.11) using the online NCBI protein Basic Local Alignment Search Tool (BLAST).
